# Classification of unilateral cervical locked facet with or without lateral mass-facet fractures and a retrospective observational study of 55 cases

**DOI:** 10.1038/s41598-021-96090-4

**Published:** 2021-08-16

**Authors:** Chao Tang, Yuan He Fan, Ye Hui Liao, Qiang Tang, Fei Ma, Qing Wang, De Jun Zhong

**Affiliations:** Department of Orthopaedics, Affiliated Hospital of Southwest Medical University, No. 25 Taiping Street, Luzhou City, 646000 China

**Keywords:** Anatomy, Diseases, Physics

## Abstract

This study describes a morphology-based unilateral cervical facet interlocking classification in an attempt to clarify the injury mechanism, instability, neurological deficits, radiological features, and determine optimum management strategies for these injuries. A total of 55 patients with unilateral cervical locked facet (UCLF) involving C3 to C7 were identified between January 1, 2012 and December 1, 2019. The injuries were classified into three types, and they were further divided into six subtypes using three-dimensional computed tomography. The injury mechanism, clinical features, neurological deficits, and imaging characteristics were analyzed, and the appropriate treatment strategies for UCLF were discussed. UCLFs were divided into the following six subtypes: UCLF without lateral mass-facet fracture (type I) in nine cases, with superior articular process fracture (type II A) in 22, with inferior articular process fracture (type II B) in seven, both superior and inferior articular process fractures (type II C) in four, with lateral mass splitting fracture (type III A) in three, and with lateral mass comminution fractures (type III B) in ten. A total of 22 (40.0%) of the 55 patients presented with radiculopathy, and 23 patients (41.8%) had spinal cord injuries. The subtype analyses showed high rates of radiculopathy in types II A (68.2%) and II C (75.0%), as well as significant spinal cord injury in types I (77.8%) and III (61.5%). Destruction of the facet capsule was observed in all patients, but the injury of disc, ligamentous complex, and vertebra had a significant difference among the types or subtypes. The instability parameters of the axial rotation angle, segmental kyphosis, and sagittal displacement showed significant differences in various types of UCLF. Closed reduction by preoperative and intraoperative general anesthesia traction was achieved in 27 patients (49.1%), and successful rate of closed reduction in type I (22.2%) was significantly lower than that in type II (51.5%) and type III (61.5%). A total of 35 of 55 patients underwent a single anterior fixation and fusion, 10 patients were treated with posterior pedicle and (or) lateral mass fixation, and combined surgery was performed in ten patients. Ten patients (18.2%) with a poor outcome were observed after first surgery. Among them, 3 patients treated with a single anterior surgery had persistent or aggravated radiculopathy and posterior approach surgery with ipsilateral facet resection, foramen enlargement, and pedicle and (or) lateral mass screw fixation was performed immediately, 5 patients treated with a short-segment posterior surgery showed mild late kyphosis deformity, and 2 patients with vertebral malalignment were encountered after anterior single-level fusion during the follow-up. This retrospective study indicated that UCLF is a rotationally unstable cervical spine injury. The classification proposed in this study will contribute to understanding the injury mechanism, radiological characteristics, and neurological deficits in various types of UCLF, which will help the surgeons to evaluate the preoperative closed reduction and guide the selection of surgical approach and fusion segment.

## Introduction

Unilateral cervical lateral mass-facet injury is a subgroup of subaxial cervical spine fractures, which accounts for 6% to 43.7% of injuries of the cervical spine^1,2^. These fractures are generally the result of hyperflexion, lateral compression, and rotation of the cervical spine due to motor vehicle accidents; and they may extend rostrally or caudally into either one of the adjacent facets, ventrally into the foramen transversarium, transverse process, and pedicle, or dorsally into the lamina^3^. With the recent progress in imaging technologies, an increasing number of cervical unilateral lateral mass-facet injuries are detected clinically. To better understand the injury mechanism, fracture pattern, and treatment strategy, several various classifications of these injuries have been presented in previous literatures^4–8^. Unilateral cervical facet dislocation caused by a complex injury is an unstable injury of unilateral lateral mass-facet fractures and generally operative management is recommended. Facet dislocation has been described as locked facet, perched facet, jumped facet, and rotational facet injuries^9^. Unilateral cervical locked facet (UCLF), a subtype of unilateral facet dislocation, has often been confused with unilateral facet dislocation in previous literatures^10,11^. Moreover, this considerable confusion is not conducive to the understanding of the mechanism of UCLF and the choice of optimal treatment strategy.

UCLF, a special type of unilateral facet dislocation, should receive more attention and be studied further for the reasons that follow. First, the mechanism of injury of unilateral facet dislocation still remains unclear^9,12–15^, and it is unknown whether there is a more complex injury mechanism of UCLF. Second, UCLF may be more debilitating than that without a locked facet because the former is more likely to cause injury to the nerve root and spinal cord^15^. Third, the spine may be in a stable state while the facets are locked unilaterally; however, the motion segment becomes overtly unstable after the locked facet is reduced, and surgical fixation and/or fusion generally need to be considered^15^. However, there is a lack of systematic description of the clinical and image characteristics and optimum management strategies for UCLF.

In this retrospective study, the patients with UCLF were classified into three types based on the injury morphologies of lateral mass-facets using the three-dimensional computed tomography. Mechanism of injury, initial spinal instability, associated lesions, and neurological deficits were analyzed. In addition, the effects of skull traction, including bedside and general anesthesia traction, on the closed reduction of UCLF, as well as the effects of surgical approaches on the clinical outcomes were discussed.

## Methods

From January 1, 2012 to December 1, 2019, 55 patients with UCLF were reviewed retrospectively. Table 1 provides the demographic data of these identified patients. In the present study, UCLF was defined as the location of the unilateral superior articular process of the injured segment behind the inferior articular process or part of the lateral mass, or unilateral inferior articular process of the injured segment located in front of the superior articular process or part of the lateral mass. All of these patients were treated by skull traction before the operation, and they underwent surgical fixation and fusion using the anterior approach, posterior approach, or combined approach. The surgical treatments were performed with an average of 6.7 days after admission (range 1–34 days). The surgical procedures were conducted at an average period of 12.0 days after injury (range 2–65 days). We confirm that all methods were performed in accordance with the relevant guidelines and regulations.

**Table 1:** The demographic data of the patients with UCLF.

Variabes	Total (%)
Sex (M: F)	47:8
Mean age (yr)	48.4 ± 11.4
Cause of injury	
Fall	29 (52.7)
MVA	11 (20.0)
Slip down	8 (14.5)
Object struck	4 (7.3)
Other causes	3 (5.5)
Site of asserted injury	
C3-4	16 (29.1)
C4-5	13 (23.6)
C5-6	14 (25.5)
C6-7	12 (21.8)
Side of asserted locked facet	
Right side	38 (69.1)
Left side	17 (30.9)
Surgical treatment after admission (d)	6.7 ± 5.7
Surgical treatment after injury (d)	12 ± 13.9

MVA, motor vehicle accident; M, male; F, female.

### Neurological and radiological assessments

Neurological function was evaluated using the American Spinal Injury Association (ASIA) impairment scale for a spinal cord injury, and descriptive recording was performed for radiculopathy.

**Figure 1:**
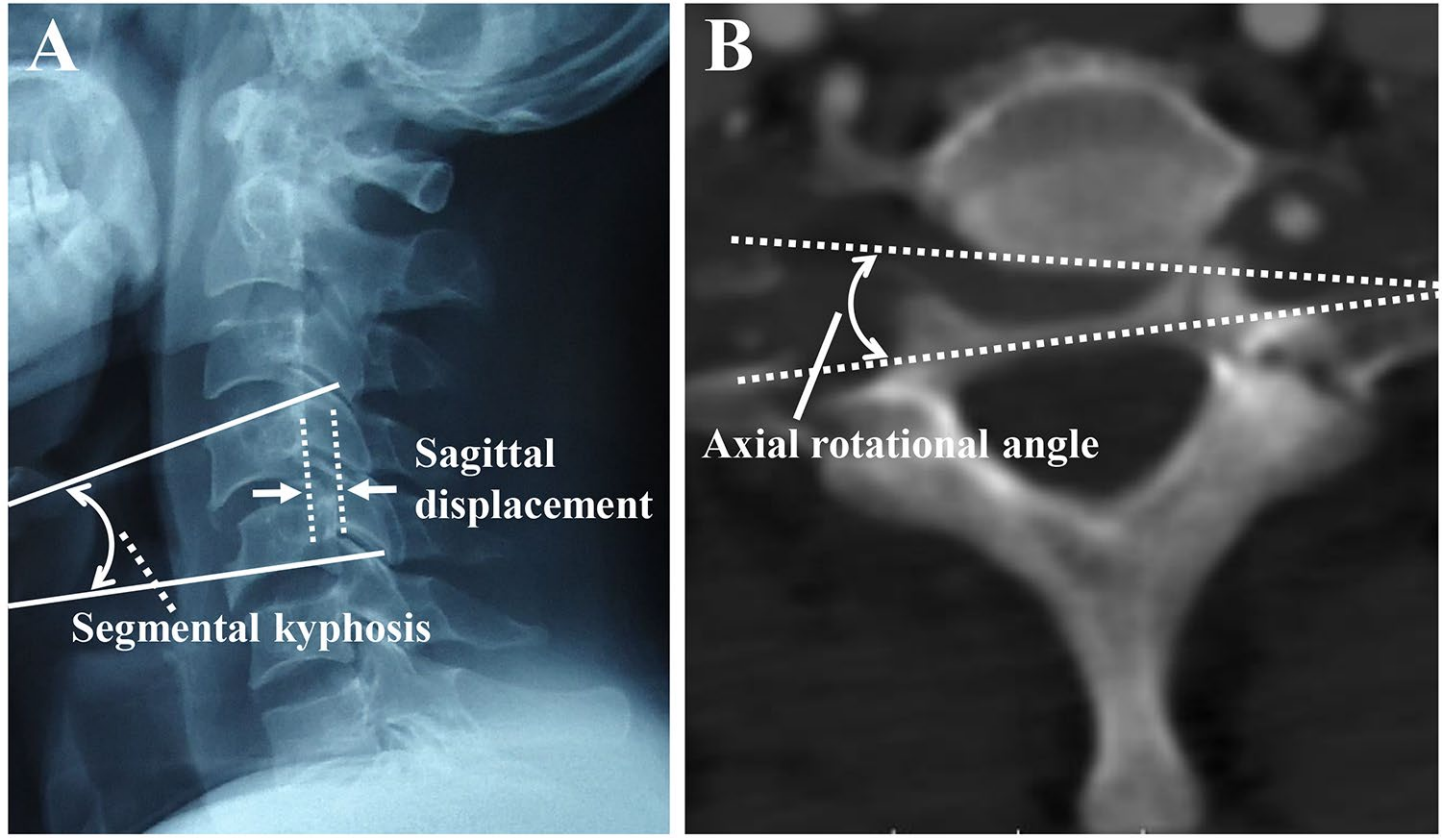
Segmental kyphosis was measured by drawing lines at a tangent to the superior margin of the cephalic vertebral body of the injured segment and a tangent to the inferior margin of the caudal vertebral body of the injured segment on the lateral view. Segmental kyphosis was classified as the angle defined by the intersection of the lines from the adjacent bodies; an extension angle is by convention negative and a flexion angle is positive. Sagittal displacement was classified as the horizontal displacement (white arrow) of the cephalic vertebra relation to the caudal vertebral body of the injured segment (**A**). Axial rotational angle was measured by drawing a line at a tangent to the two most posterior points of the two adjacent vertebral bodies observed by axial computed tomography (**B**).

The instability parameters of sagittal displacement and segmental kyphosis were measured on a neutral lateral X-ray (Fig. 1A), and the axial rotational angle was measured on CT (Fig. 1B). An X-ray (C-arm) was used to evaluate the closed reduction of locked lateral mass-facet under skull traction. Three-dimensional computed tomography (3D-CT) allowed identification of fractures in the injury segment and adjacent cervical bone structure, thereby confirming the associated cervical spine fractures. Based on the magnetic resonance imaging (MRI) analysis, the signal changes or rupture of the intervertebral disc and anterior longitudinal ligaments (ALL), posterior longitudinal ligament (PLL), and facet capsule were examined mainly in the injured segment. Initial instability was defined by White as follows: > 3.5 mm displacement, > 11° kyphosis of injury segment on static or dynamic X-ray films, or a > 10° rotational difference from the injury segment on CT.

The spinal alignment and fusion status were evaluated using static, dynamic X-ray films, and CT scans during the follow-up. A successful outcome in this series necessitated an anatomic restoration of the spinal column, no requirement for secondary surgery, an improvement in the pain or neurologic deficits, solid fusion of the segment, no adjacent instability, and no late kyphosis or translation (> 11° and 3.5 mm) during the follow-up.

In order to evaluate inter- and intraobserver reliabilities for this new classification, a designer team (authors of this study) selected CT scan images of 31 cases (6 cases of type I, IIA, IIB, IIIB, 4 cases of type IIC, and 3 cases of type IIIA). All cases derived from our database of 55 UCLF patients from January 1, 2012 to December 1, 2019 in our institution. We identified 3 readers to evaluate these CT scan images; they were all orthopedic physicians (observer 1: C. T. MD, observer 2: Y. L. MD, observer 3: Q. T. MD) who were trained in spine surgery for more than 6 years in our institution. The CT images were completed independently by 3 readers in PACS (picture archiving and communication system) software workstation. The same questionnaire was sent to the readers in an aleatory order 2 weeks after the first reading to analyze the intraobserver agreement. The graded data was collected and analyzed for reliability; intra- and interobserver agreements were assessed for each type of the classification.

### Statistical analyses

SPSS software version 19.0 (SPSS Inc, Chicago, IL, USA) was used for all statistical analyses. We evaluated the inter-observer agreement through the calculation of the weighted kappa coefficient for each pair of judges (readers). To evaluate the degree of intra-observer agreement, we calculated the weighted kappa coefficient according to the same weighting matrix as for the degree of agreement between the different evaluators. Descriptive statistics were applied to the epidemiologic data. All of the data, including the coronal and sagittal angles and sagittal vertebral displacement, were analyzed using the Student’s *t* test in the parametric value. The Chi-square test or Fisher exact test was used to compare the categorical variables. The level of significance was set at *p* < 0.05. An analysis of variance and the Duncan multiple comparison test, with α = 0.05, were used to determine the differences between the various types or subtypes.

### Ethical approval and consent to participate

The manuscript submitted does not contain information about medical device(s)/drug(s). And the study was approved from the Ethics Committee of the Affiliated Hospital of Southwest Medical University, China (Ethical parameters. KY2020165).

### Informed consent

The informed consent of this retrospective study is waived by the Ethics Committee of the Affiliated Hospital of Southwest Medical University, China.

## Results

**Figure 2:**
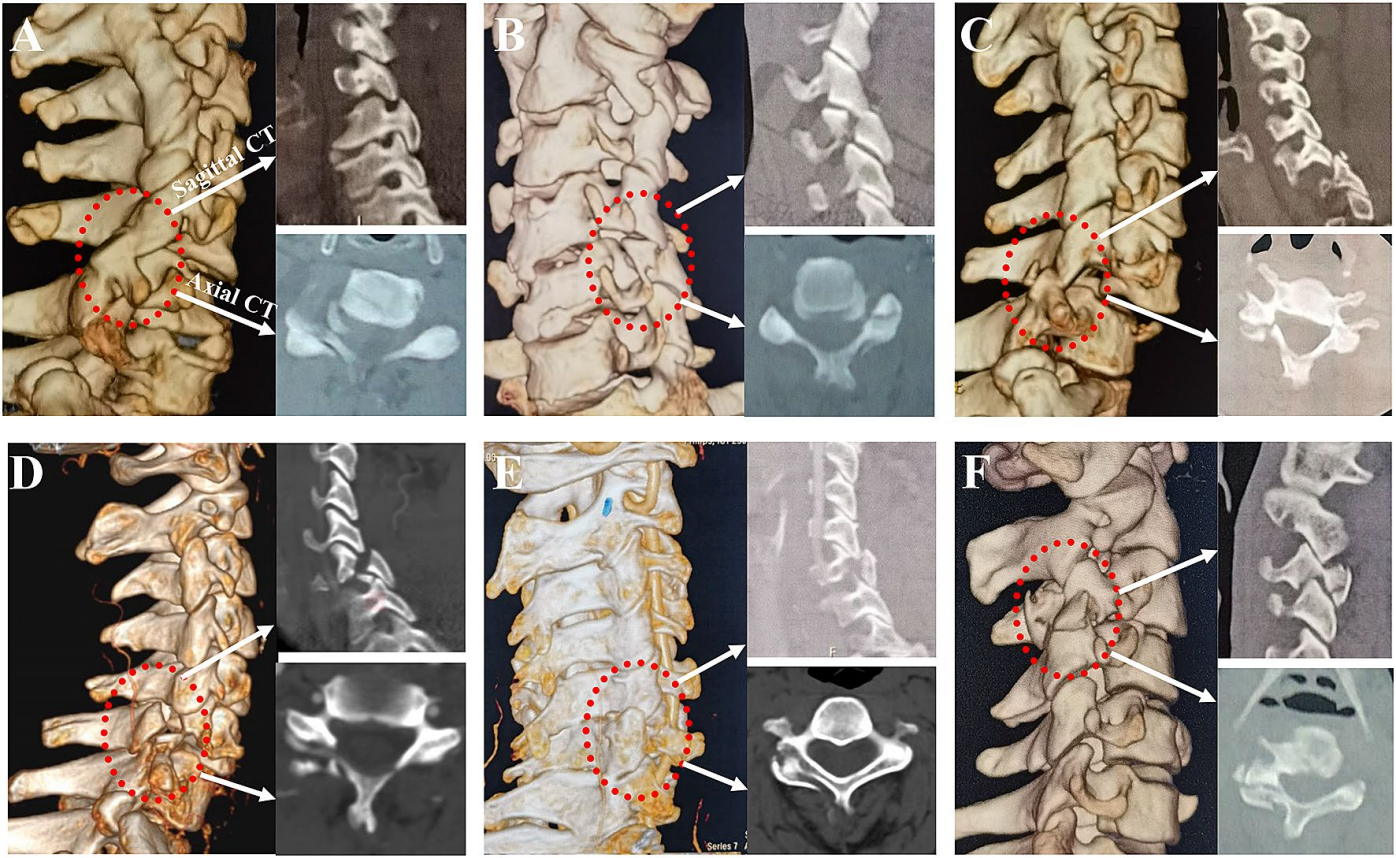
Description of the classification of UCLF. *Type I:* defined as unilateral cervical locked facet without lateral mass-facet fracture (**A**); *Type II A*: unilateral cervical locked facet with ipsilateral superior articular process fracture of the injury segment (**B**); *Type II B*: unilateral cervical locked facet with ipsilateral inferior articular process fracture (**C**); *Type II C*: unilateral cervical locked facet with ipsilateral both superior and inferior articular process fractures (**D**); *Type III A:* unilateral cervical locked facet with ipsilateral lateral mass splitting fracture (**E**); *Type III B:* l unilateral cervical locked facet with ipsilateral lateral mass comminuted fractures (**F**).

The classification of UCLF was described based on the following three criteria of injury morphology: (1) unilateral locked facet without lateral mass-facet fracture, (2) unilateral locked facet with ipsilateral facet fractures, and (3) unilateral locked facet with ipsilateral lateral mass fractures. Three authors classified the patients into one of the three types of UCLF according to the axial CT and three-dimensional reconstruction appearances, which were as follows:

*Type I:* UCLF without lateral mass-facet fracture (Fig. 2A).

*Type II:* UCLF with ipsilateral facet fractures. This fracture pattern showed a locked facet with articular process fractures, but without lateral mass fractures. Type II injuries were further divided into the following three subtypes according to the site of the facet fractures:

*II A*─superior articular process fracture of the injury segment. The bony fragments were pushed forward by the inferior articular process and generally invaded the foramen (Fig. 2B).

*II B*─inferior articular process fracture of the injury segment. The bony fragments were pushed backward by the superior articular process and generally did not invade the foramen (Fig. 2C).

*II C*─both superior and inferior articular process fractures of the injury segment. The bony fragments of the superior articular process fracture generally invaded the foramen (Fig. 2D).

*Type III:* UCLF with ipsilateral lateral mass fractures. This fracture pattern showed a locked facet with lateral mass fractures, and it may be accompanied by the fractures of the superior and (or) inferior articular processes. Type III injuries were further divided into the following two subtypes according to the fracture pattern:

*III A*─lateral mass splitting fracture. This fracture pattern had a vertical fracture of the lateral mass on the coronal plane, creating anterior–posterior separation with invagination of the superior (or) inferior articular process of the caudal (or) cephalic adjacent vertebra (Fig. 2E).

*III B*─lateral mass comminuted fractures. This fracture pattern had multiple fracture lines in the unilateral lateral mass, resulting in the inferior articular process or part of the lateral mass of the injured segment being located in front of the superior articular process (Fig. 2F).

Among the 55 patients with UCLF, type I, type II, and type III injury patterns were observed in nine cases, 33 cases, and 13 cases, respectively. According to the classification, there were 22 cases of type II A, seven cases of type II B, and four cases of type II C among patients with type II injury pattern. There were three cases of type III A and ten cases of type III B among patients with type III injury pattern.

The interobserver reliability according to different types of UCLF based on CT scan images is presented in Table 2; almost perfect agreement was observed in both the first evaluation with 0.85 (95% CI 0.82–0.88) and the second with 0.90 (95% CI 0.87–0.92). The intra-observer analysis is presented in Table 3 and showed an almost perfect agreement among readers with к = 0.89 (95% CI 0.75–0.92).

**Table 2:** Inter-observer reliability according to various types.

Type of UCLF	First reading Fleiss’s κ (95% CI)	Second reading Fleiss’s κ (95% CI)
Type I	1	1
Type IIA	0.86	0.90
Type IIB	0.85	0.89
Type IIC	0.83	0.91
Type IIIA	0.78	0.89
Type IIIB	0.81	0.86
Overall	0.85 (0.82–0.88)	0.90 (0.87–0.92)

**Table 3:** Cohen’s κ for intra-observer reliability.

Observer	Cohen’s κ	95% CI	
		Lower bound	Upper bound
1	0.89	0.74	0.96
2	0.86	0.71	0.91
3	0.91	0.81	0.98
Overall	0.89	0.75	0.92

### Neurological findings

Table 4 summarizes the neurologic status and radiologic results of each type and subtype at admission. A total of 22 patients (40.0%) were presented with radiculopathy on admission. Type II had the highest (57.6%) prevalence of radiculopathy among the three types, and subtype analysis showed that the prevalence of radiculopathy in type II B (14.3%) was significantly lower than that in type II A (68.2%) and type II C (75.0%). A total of 23 patients (41.8%) had spinal cord injuries; seven cases of type I (ASIA A-4, ASIA B-2, and ASIA C-1), four cases of type II A (ASIA A-1, ASIA C-2, and ASIA D-1), three cases of type IIB (ASIA A-1, ASIA C-1, and ASIA D-1), one case of type II C with D grade of ASIA scale, two cases of type III A (ASIA A-1 and AISA C-1), and six cases of type III B (ASIA C-1 and ASIA D-5).

**Table 4:** Initial neurological status, intervertebral disc and ligamentous injury on the magnetic resonance images according to each type (or) subtype of classification in patients with UCLF.

Injury type	Neurological Injury (%)		Associated Soft Tissue Injury (%)				Associated cervical spine fracture (%)		Traumatic cervical degenerative disease (%)
	Radiculopathy	Spinal cord injury	Disc	ALL	PLL	Facet capsule	Vertebra fx	Appendix fx	
Type I (n = 9)	1 (11.1)	7 (77.8)*	9 (100)*	9 (100)*	9 (100)*	9 (100)	7 (77.8)*	5 (55.6)	3 (33.3)
Type II (n = 33)	19 (57.6)*	8 (24.2)	28 (84.8)	15 (45.5)	23 (69.7)	33 (100)	21 (63.6)	7 (21.2)*	10 (30.3)
IIA (n = 22)	15 (68.2)	4 (18.2)	20 (90.9)	6 (27.3)^#^	19 (86.4)^#^	22 (100)	18 (81.8)^#^	2 (9.1)^#^	8 (36.4)
IIB (n = 7)	1 (14.3)^#^	3 (42.9)^#^	6 (85.7)	6 (85.7)	2 (28.6)^#^	7 (100)	2 (28.6)	3 (42.9)	1 (14.3)^#^
IIC (n = 4)	3 (75.0)	1 (25.0)	2 (50.0)^#^	3 (75.0)	2 (50.0)	4 (100)	1 (25.0)	2 (50.0)	1 (25.0)
Type III (n = 13)	2 (15.4)	8 (61.5)	11 (84.6)	5 (38.5)	4 (30.8)*	13 (100)	5 (38.5)*	9 (69.2)*	1 (7.7)*
IIIA (n = 3)	0	2 (66.7)	2 (66.7)	1 (33.3)	1 (33.3)	3 (100)	1 (33.3)	2 (66.7)	0
IIIB (n = 10)	2 (20.0)	6 (60.0)	9 (90.0)	4 (40.0)	3 (30.0)	10 (100)	4 (40.0)	7 (70.0)	1 (10.0)

ALL, anterior longitudinal ligament; PLL, posterior longitudinal ligament.

*Indicates a significant difference among the three types of UCLF based on the Chi-square test, *p* < 0.05.

^#^Indicates a significant difference among the three subtypes of type II based on the Fisher exact test, *p* < 0.05.

### Radiographic features

In terms of an associated soft tissue injury, 48 patients, 29 patients, 36 patients, and 55 patients had an injury to the disc, ALL, PLL, and facet capsules, respectively. Fourteen (25.5%) of these 55 patients had a traumatic cervical degenerative disease. In terms of an associated cervical spine fracture, 33 patients (60.0%) had a single or multiple cervical vertebral fracture, and 21 patients (38.2%) had appendix fractures of the cervical vertebra. Type I had a significant higher prevalence of associated soft tissue injury and vertebral fracture, followed by type II and type III. And the prevalence of injury to the PLL in type II A was significantly higher than that in type II B and type II C, but it was significantly lower than that to the ALL. The patients with type III had a significantly lower percentage (7.7%) of traumatic cervical degenerative disease than that in type I (33.3%) and II (30.3%) (Table 4).

**Figure 3:**
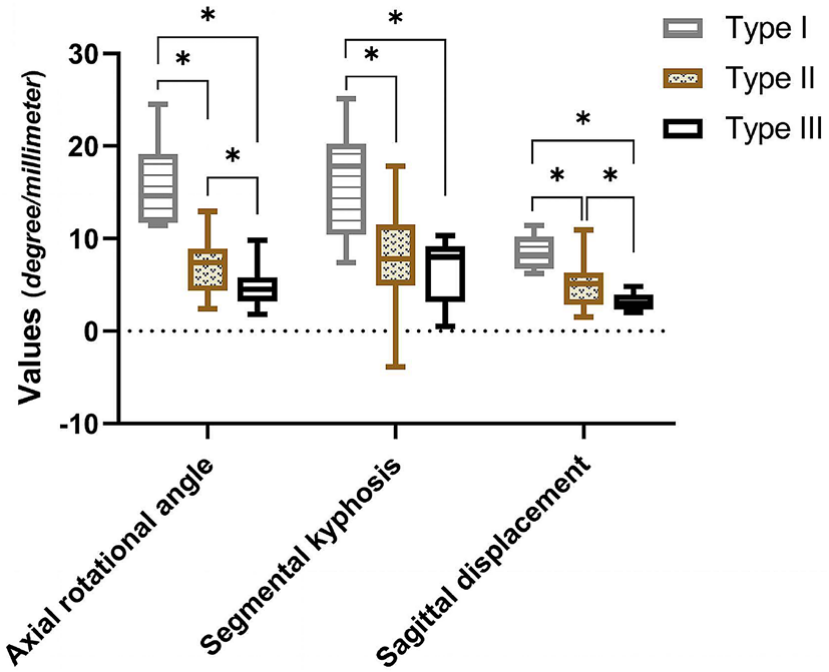
Box and whisker plots showing the instability parameters of the injured segment in each type of UCLF. Statistical significances were determined by One-way ANOVA analysis of variances between the groups, * indicates a significant difference between the types based on Duncan's multiple comparison tests.

In these 55 patients, the mean axial rotation angle, segmental kyphosis, and sagittal displacement on admission were 7.9° ± 4.7°, 8.8° ± 6.1°, and 5.1 mm ± 2.6 mm, respectively. All of these patients had an unstable cervical spine based on the instability criteria. Analysis of variance showed that there were statistically significant differences in the parameters of instability among the three types (Fig. 3).

### Overall management of these 55 patients

Closed reduction of the locked facet was evaluated in all patients by preoperative and intraoperative general anesthesia traction. The weights of preoperative traction, starting from 3 kg, were gradually (1 kg per day) and equally added to a total of 6 kg. Intraoperative general anesthesia traction weights were performed from 4 kg for 3 min; the traction weight was increased in accordance with the reduction in patients with UCLF but it was no more than one-sixth to one-fifth of the patient’s weight, and somatosensory evoked potentials (SEPs) were used to monitor the neurologic signal throughout the traction procedure. The mean weights in preoperative and intraoperative general anesthesia traction were 4.5 kg and 8.6 kg, respectively.

Table 5 summarizes the results of management of each type (or) subtype of patients with UCLF. Closed reduction was achieved in 27 (49.1%) of these 55 patients; 18 patients achieved successful reduction with preoperative traction, and 9 patients achieved successful reduction with intraoperative general anesthesia traction. Successful rate of closed reduction in type III (61.5%) was significantly higher than that in type II (51.5%) and type I (22.2%).

**Table 5:** The management of each type or subtype patients with UCLF.

Injury pattern	Closed reduction by skull traction			Surgical management		
	Pre-O	General anesthesia	Total (%)	Anterior	Posterior	Combined
Type I (n = 9)	0	2	2 (22.2)*	2	3	4
Type II (n = 33)	13	4	17 (51.5)	25	4	4
IIA (n = 22)	8	2	10 (45.5)	16	3	3
IIB (n = 7)	4	1	5 (71.4)^#^	6	1	0
IIC (n = 4)	1	1	2 (50.0)	3	0	1
Type III (n = 13)	5	3	8 (61.5)*	8	3	2
IIIA (n = 3)	1	1	2 (66.7)	2	0	1
IIIB (n = 10)	4	2	6 (60.0)	6	3	1

*Indicates a significant difference among the three types of UCLF based on the Chi-square test, *p* < 0.05.

^#^Indicates a significant difference among the three subtypes of type II based on the Fisher exact test, *p* < 0.05.

Thirty-five of these 55 patients were treated with the anterior cervical approach surgery alone; a single-level anterior cervical discectomy and fusion (ACDF) was performed in 14 patients, 17 patients were treated with a two-level fusion of ACDF or anterior cervical corpectomy and fusion (ACCF), and 4 patients were treated with a three-level fusion of ACDF combined with ACCF. Ten of these 55 patients were treated with posterior lateral mass-facet release and reduction, and pedicle and (or) lateral mass screw fixation (4 cases with a three-level fixation, 3 cases with a two-level fixation, and 3 cases with a single level fixation). Combined posterior and anterior approach surgery was performed in 10 patients; a single-level fixation and fusion was performed in 4 cases, two-level fixation and fusion was performed in 5 cases, and three-level fixation and fusion was performed in 1 case.

### Clinical and radiologic outcomes

Arthrodesis was successfully performed in all cases. In total, 40 patients have been followed up to date, 13 patients were lost to follow-up during the final follow-up at more than 12 months, and 2 patients died due to complications of severe spinal cord injury at the 13- and 16-month follow-up. The mean follow-up was 23.5 months (range 12–60 months).

Nineteen of the 22 radiculopathy cases recovered from radicular pain and weakness in the upper extremities after closed reduction or surgical treatment, and 3 patients (2 cases of type III B and 1 case of type II A) treated with a single anterior surgery had persistent radiculopathy and posterior approach surgery with ipsilateral facet resection, foramen enlargement, and pedicle and (or) lateral mass screw fixation was performed immediately. Neurologic improvement of more than one grade was observed in 19 of the 23 myelopathy cases at the 12-month follow-up.

**Figure 4:**
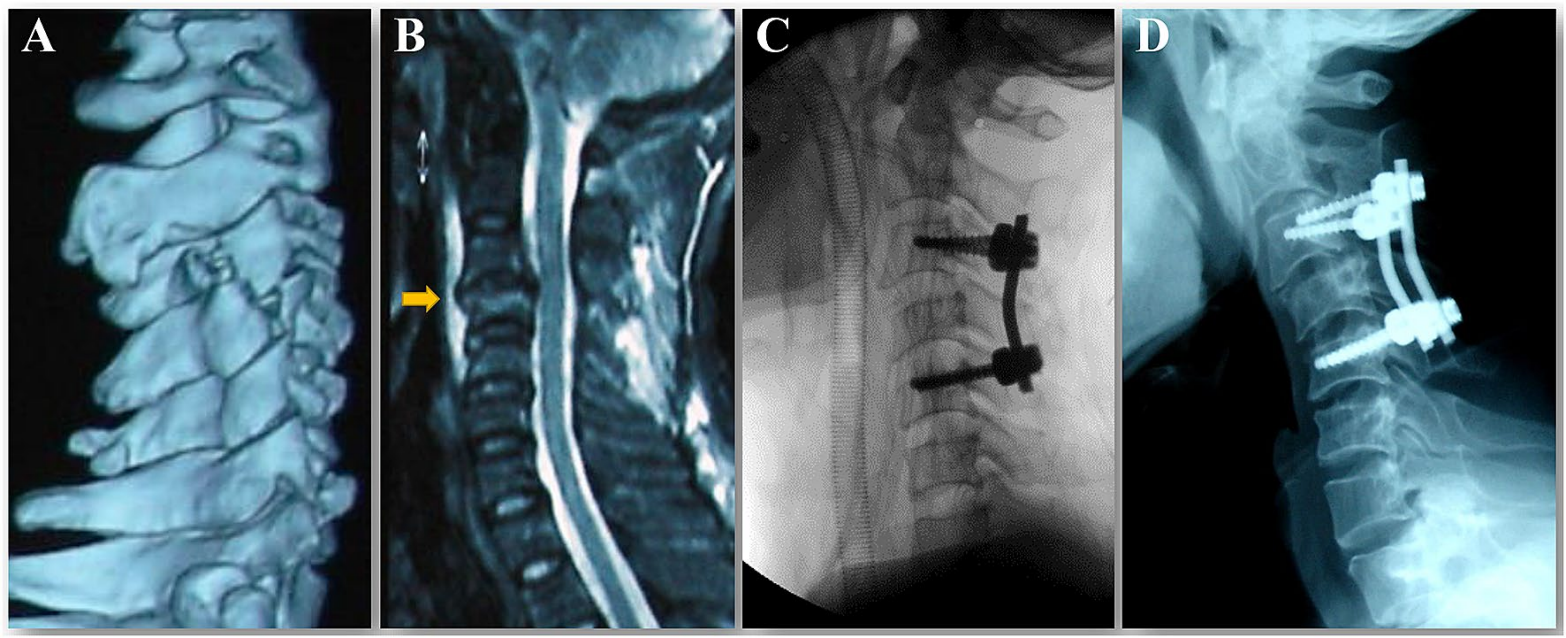
A 39-year-old man who was diagnosed as type IIIA injury pattern of UCLF. Preoperative CT three-dimensional reconstruction showed that a vertical fracture of the C4 right lateral mass created anterior–posterior separation with invagination of the inferior articular process of the cephalic adjacent vertebra (**A**). Cervical vertebral body compression fracture, disc and anterior longitudinal ligament injury, and hematoma in the prevertebral space (yellow arrow) were discovered on sagittal T2-weighted MR image (**B**). The patient underwent posterior facet release and reduction, and C3-5 pedicle screw fixation (**C**). A mild late kyphosis deformity because of intervertebral space subsidence and vertebral collapse was found at 8 months follow-up after surgery (**D**).

**Figure 5:**
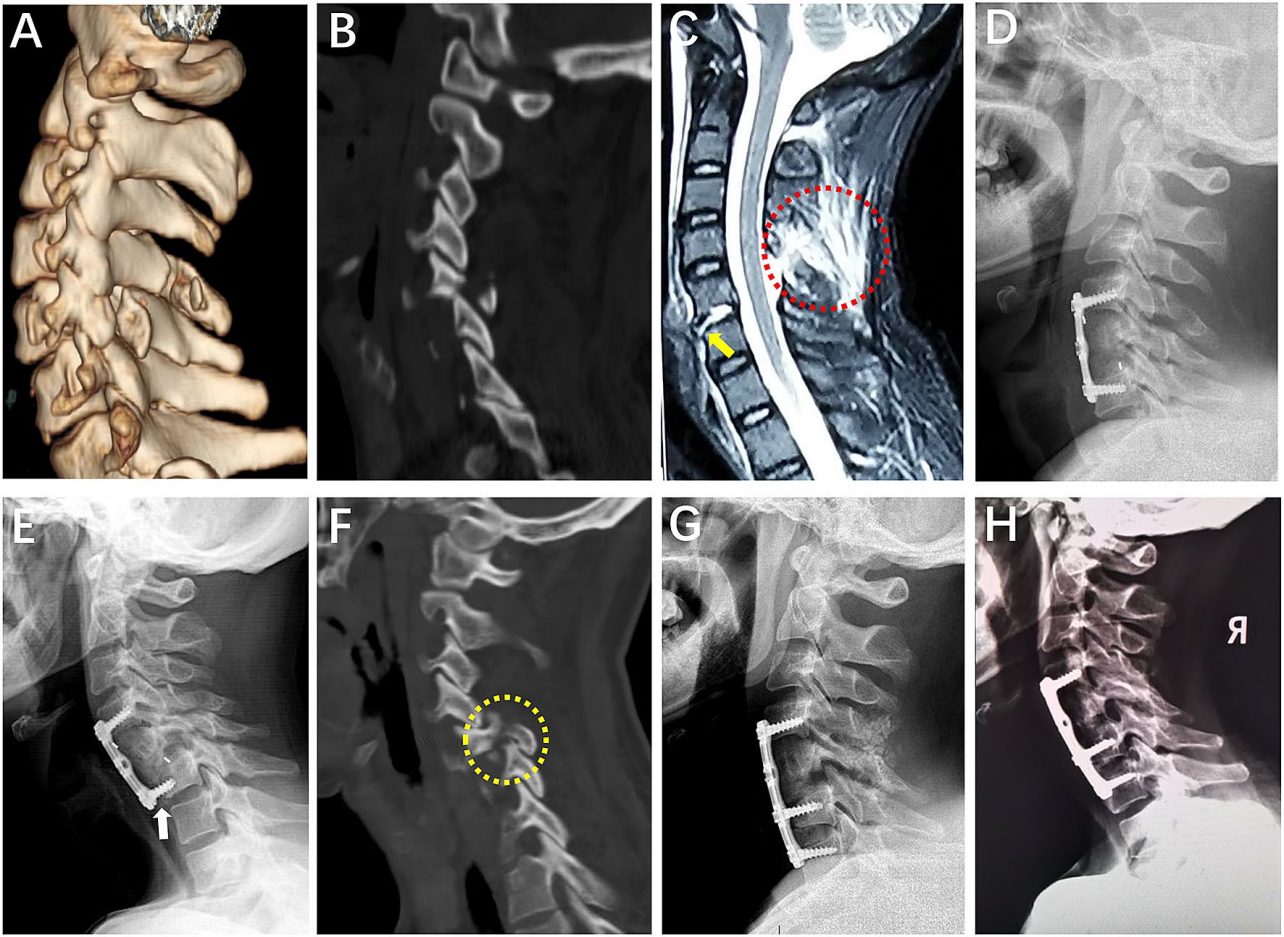
A 40-year-old man who was diagnosed as type IIB injury pattern of UCLF. Left side facet interlocking with ipsilateral inferior articular process fracture at C5-6 motion segment was discovered on CT three-dimensional reconstruction appearance (**A**, **B**). Sagittal T2-weighted MR image showed that the patient had a rupture of anterior longitudinal ligament and intervertebral disc (yellow arrow), and severe posterior ligament complex injury (red circle) (**C**). The patients had a successful closed reduction by preoperative skull traction, and anterior C5 corpectomy and fusion was performed (**D**). At 4 months follow-up after surgery, the patient had a sever neck pain and dysphagia due to failed reduction with migration of screws (**E**) and facet dislocation (yellow circle) at the injury segment (**F**). A revision surgery was performed by posterior facet release, reduction and autogenous bone fusion combined with extending the anterior fusion segment (**G**). The cervical spinal alignment was stable and the graft fusion was satisfactory at 20 months follow-up after revision surgery (**H**).

The follow-up radiographic analysis showed that seven patients had a poor radiographic outcome. Among them, five cases (2 cases of type III B and 1 case of type II A, II B, and III A respectively) treated with a single short-segment posterior pedicle and (or) lateral mass screw fixation showed mild late kyphosis deformity because of intervertebral space subsidence and (or) vertebral collapse (Fig. 4), and one case of cephalad vertebra malalignment was encountered as the patient had adjacent disc and appendix injury while a single level ACDF was performed. Although a slight deformity or malalignment was observed in six cases, there was no obvious neck pain, radiculopathy, or myelopathy symptom, and revision surgery was not required in these patients. Unfortunately, one case that underwent C5 ACCF had severe neck pain and dysphagia due to failed reduction with facet dislocation and migration of screws in the injury segment at the 4-month follow-up, and revision surgery was performed (Fig. 5). Thus, among these 55 patients, 10 patients (18.2%) had a poor outcome based on the clinical and radiographic analyses after surgery and their detailed data are summarized in Table 6.

**Table 6:** The detailed data of poor outcome cases and management.

No	Sex/age (y)	Injury pattern	Injury level	Neurological injury		Associated soft tissue injury				Associated cervical spine fx		Surgical management	Poor outcome	Treatment
				Radiculopathy	Spinal cord injury	Disc	ALL	PLL	Facet capsule	Vertebra fx	Appendix fx			
1	M/58	Type IIA	C5-6	Y	N	Y	Y	Y	Y	Y	N	C5-6 ACDF	NC by FF	Revision surgery^¶^
2	M/59	Type IIA	C6-7	Y	N	Y	Y	Y	Y	Y	Y	C6-7 ACDF	NC by FF	Revision surgery^¶^
3	M/36	Type IIIB	C5-6	Y	N	Y	Y	Y	Y	Y	N	C6 ACCF	NC by FF	Revision surgery^¶^
4	F/40	Type IIIB	C3-4	N	ASIA: D	Y	Y	Y	Y	Y	Y	C2-4 PSF/LMSF	Kyphosis	NA^#^
5	F/78	Type IIA	C5-6	Y	N	Y	Y	N	Y	Y	Y	C4-7 LMSF	Kyphosis	NA^#^
6	M/41	Type IIB	C3-4	N	ASIA: C	Y	Y	Y	Y	Y/C1-2 RD	Y	C1-4 PSF	Kyphosis	NA^#^
7	M/39	Type IIIA	C3-4	N	ASIA: D	Y	Y	N	Y	Y	Y	C3-5 PSF	Kyphosis	NA^#^
8	M/48	Type IIB	C5-6	N	ASIA: A	Y	Y	Y	Y	Y	N	C5-7 PSF/LMSF	Kyphosis	NA^#^
9	M/44	Type IIA	C6-7	Y	N	Y	Y	Y	Y	Y	N	C6-7 ACDF	Adjacent instability	NA^#^
10	M/40	Type IIB	C5-6	N	ASIA: D	Y	Y	N	Y	Y	Y	C5 ACCF	Failed reduction migration of screws	Revision surgery^†^

ASIA, American Spinal Injury Association; RD, rotational dislocation; ACDF, anterior cervical discectomy and fusion; ACCF, anterior cervical corpectomy and fusion; PSF, pedicle screw fixation; LMSF, lateral mass screw fixation; NC, never root compression; FF, fracture fragment; NA, not available.

^#^No revision surgery.

^¶^Revision surgery by posterior ipsilateral facet resection, foramen enlargement, and pedicle and (or) lateral mass screw fixation.

^†^Revision surgery by posterior facet release and reduction combined with extending the anterior fusion segment.

## Discussion

UCLF, a subtype of the cervical facet injuries, is significantly different from the other types of unilateral lateral mass-facet injury. The presence of locked facet not only suggests an injury caused by significant energy, but also a mechanism of flexion distraction^16^, and the patients with locked facet usually present with a worse neurological deficit^17^. Although there is no current universally accepted classification of UCLF, Sun et al. reported a classification of unilateral lateral mass-facet fractures based on the location of injury and fracture pattern, but a locked facet may be found in different types of the classification^7^. However, a locked facet along with subluxation, perched facet was classified as one same subtype in the AO Spine subaxial cervical spine injury classification system by Vaccaro et al.^8^. And the previous classification system did not pay attention to the success rate of closed reduction in various types of patients. At the same time, there are few discussions on the choice of surgical approaches and fusion segments for each type. To better understand the injury mechanism and clinical characteristics of UCLF and to further guide the surgical treatment strategies, we first described the classification of UCLF according to the injury pattern using the 3D-CT.

### Classification of UCLF and injury mechanism and radiologic instability

The injury mechanism of unilateral cervical facet dislocation has been attributed to combined flexion and rotation^18–20^, but whether flexion or rotation is a component of greater importance in unilateral facet interlocking remains unclear. In the present study, seven (77.8%) of nine patients with type I had a prominent lower vertebral compression fracture or burst fracture in the injured motion segment. Allen^14^ and Vaccaro^8^ considered that the injury mechanism of compression vertebral fracture is compressive or distractive flexion. However, we found that the intervertebral disc, ALL, and PLL ruptures were observed in all patients with type I. Some authors deemed that ALL failure occurred in tension and (or) shear injury but not in compression flexion^21,22^. Thus, rotation injury, as well as flexion injury, is an important component in patients with type I, and a powerful rotational shear force leads to disc and ligament injury and vertebral axial rotation. The mean axial rotational degree was 15.6° in type I, which was larger than that in type II (7.1°) and III (4.7°), and the result also confirms that the patients with type I have the greatest rotational instability among patients of UCLF. Crawford^15^ and Caravaggi^16^ had successfully created a model of UCLF without lateral mass-facet fracture in cadaveric specimens by the experimental process of an anteriorly directed force and adequate torque of axial rotation, followed by a smaller lateral bending force. These experiments seem to confirm our notion.

However, on the contrary, if axial rotation and lateral force occur first and dominate in the process of injury, and they are followed by a smaller flexion load, it may lead to the type III injury pattern of UCLF. The lateral mass-facet of the locked side is the major injury vector when rotational and lateral tilt components occur, and it is prone to split or comminuted fracture under the stresses. Lateral appendix fractures could be found at the same time. A total of nine (69.2%) of the 13 patients had ipsilateral appendix fracture, but a mild compression vertebral fracture and PLL rupture in the injured motion segment were observed only in five (38.5%) patients and four (30.8%) patients, respectively. It is worth noting that the axial rotational degree of the vertebra is not large as the rotation force could be reduced by the reverse obstructed force of the facet. For the type II injury pattern, the mechanism of injury may be complex and diverse. According to the research data, the axial rotational angle in patients with type II was smaller than that in type I, and it was observed in only seven (21.2%) of the 33 patients with appendix fractures. These data indicated that the rotation and lateral tilt force may be mild and small. However, a higher prevalence (69.7%, 23/33) of PLL injury but lower prevalence (45.5%, 15/33) of ALL injury was observed in patients with type II, especially in type II A (86.4% in PLL, 27.3% in ALL). And 18 (77.8%) of 22 patients with type II A had a prominent lower vertebral compression fracture or burst fracture in the injured motion segment. The characteristics of those ligament injuries and vertebral fractures are consistent with hyperflexion injuries. According to Allen’s classification of lower cervical spine injury, the distractive flexion stage 2 (DFS2) lesion is a unilateral facet dislocation, and a small bony fleck may be displaced from the posterior surface of the articular process, which is displaced forward^14^. It is noticeable that this fracture pattern of the facet in DFS2 is similar to type II A in our classification. On the other hand, some authors have attributed unilateral articular process factures primarily to a hyperextension or hyperextension combined with a rotational injury mechanism^6,23,24^. In this study, more than 70% of patients with types II B and II C had no obvious vertebral compression fracture in the injury motion segment, but they had a serious ALL injury. The characteristics of injury pattern in types II B and II C are not consistent for hyperflexion injury, but seems to be suitable for hyperextension injury. The unilateral articular process is the major injury vector when hyperextension combined with a rotational injury occur; superior and (or) inferior articular process fractures with locked facet may be observed, and they are frequently accompanied by spinous process and (or) lamina fractures and splitting fracture of the posterior edge of the vertebral body in this injury mechanism.

### Classification of UCLF and neurological implications

Andreshak et al. included 308 patients with unilateral facet dislocation through a literature review, and they reported that 116 (37.7%) patients had incomplete or complete spinal cord injuries and 114 (37.0%) patients had a nerve root deficit^9^. In our study, 22 (40.0%) of the 55 patients presented with radiculopathy on admission and 23 patients (41.8%) had incomplete or complete spinal cord injuries. A total of seven (77.8%) of the nine patients with type I had a spinal cord injury, and more than 50% of them experienced complete quadriplegia. However, the incidence of spinal cord injury in types II and III is 24.2% and 61.5%, respectively, and most of the patients have an incomplete spinal cord injury with a C or D grade of the AISA scale. In clinical settings, Shanmuganathan et al. have found that unilateral locked facet without an associated fracture may be more likely to cause injury to the spinal cord than unilateral locked facet with fractures^5^. Although there was no lateral mass-facet fracture in patients with type I, the axial rotation angle in this type was largest among the three types of UCLF. Also, the degree of spinal cord injury aggravated with an increase in the axial rotation angle due to the effect of rotational instability on the spinal canal.

As shown by McLain et al., the high incidence of nerve root lesions is understandable since the neuroforamen is narrowed at some point by the dislocating facet or resulting fracture fragments^13^. According to our findings, a total of 22 patients had nerve root deficits, type II C had the highest (75.0%) incidence of nerve root lesions, followed by type II A (68.2%), type III B (20.0%), type II B (14.3%), and type I (11.1%). According to the characteristics of fracture morphology, most of the patients had nerve root compression in the neuroforamen resulting from fracture fragments of the superior articular process and (or) lateral mass.

### Closed reduction and surgical strategy in UCLF

Closed reduction in patients with UCLF is more difficult than that in patients with bilateral locked facet joints^25,26^. In the previous literature, the prevalence of success of reduction in patients with unilateral locked facet was between 14 and 36%^18,27^. Wang et al. modified a reduction procedure with the halo-vest system through bracing of multiple flexion–extension activities, and all patients with unilateral locked facet in their study achieved successful closed reduction^28^. In the present study, closed reduction by skull traction was achieved in 27 (49.1%) of the 55 patients. Among these patients with closed reduction, the highest prevalence of success of closed reduction was found in type III with a rate of 61.5%, followed by type II (51.5%) and type I (22.2%). Wilson et al. considered that the main reason for the low success rate of reduction is that the axial rotational deformity cannot be controlled by traction of the skull^18^. The success of the closed reduction is an important basis for the surgical approach of the patients in this retrospective study. In the case of successful closed reduction, the anterior fusion was selected, and if not, the posterior locked facet release reduction and internal fixation were selected. Meanwhile, the combination of anterior fusion was evaluated based on the injury of the vertebral body and the disc.

Unfortunately, this series of patients showed a high rate (18.2%) of poor outcomes. Among them, the poor outcome rate of single anterior approach surgery was 14.3% (5/35), and that of posterior approach surgery was 50.0% (5/10). Lifeso and Colucci propose that late-developing kyphosis after posterior fusion procedures was purportedly related to anterior disc space collapse despite the presence of solid posterior fusion^29^. In this study, we found that most of the patients with UCLF, especially in types I and II, had an anterior annular and ligamentous complex disruption under the rotationally unstable injury and vertebra fractures due to compressive or distractive flexion injury. Meanwhile, posterior approaches are less likely to restore cervical lordosis than anterior approaches and absence of a normal cervical alignment may have a negative influence on the long-term outcome. Thus, when a posterior cervical approach is planned, a preoperative MRI is recommended to evaluate a relevant disc, ligamentous complex, and vertebral injuries. In such cases, an anterior approach would be preferable or combined with posterior cervical approach. Therefore, according to this study, the imaging damage features and closed reduction condition of each type in UCLF classification system, it is of certain clinical significance to guide the surgical options for various type of UCLF. 1) For type I injury, closed reduction is difficult, and often combined with a severe spinal cord injury. Therefore, posterior facet release reduction and internal fixation is generally the first choice, laminoplasty and spinal canal decompression can be performed at the same time for patients with severe spinal cord injury. However, if severe vertebral fracture and traumatic disc herniation are combined at the same time, combined anterior fusion also needs to be considered. 2) For type II, the success rate of closed reduction is about 50%, and this type is often accompanied with vertebral fracture and disc injury. Therefore, anterior fusion can be the first choice, especially for type IIB. However, for type IIA, severe posterior tension band injury is common, so the combination of posterior short segment fixation or anterior long segment fusion over the injury segments can achieve satisfactory clinical results. 3) In type III injury, close reduction is easy, and the incidence of ligament complex injury is low. Therefore, anterior fusion can achieve better stability. However, for type IIIB injury, which belongs to the lateral mass comminuted fractures, the combination of posterior short segment fixtion can better maintain the stability of the cervical spine, and the bony fragments can be removed and the nerve root can be explored.

Previous literature has reported that patients treated with ACDF for unilateral facet fracture and dislocation had direct anterior decompression of neural elements^30^, lower rate of wound infection, higher rate of radiological bone union, and better cervical alignment compared to those treated with a posterior approach^31^. However, the following points should be noted in anterior surgery: First, for patients with failed closed reduction, posterior facet release or resection is optional, which is helpful for reduction of locked facet and for preventing aggravation of the spinal cord injury caused by forced anterior reduction. Second, a single anterior approach may lead to a risk of neurologic deterioration due to residual fracture fragments in the neuroforamen, and posterior surgery should be performed for ipsilateral nerve root canal decompression. Third, for selection of the anterior fusion segment, some authors have insisted that facet fractures need to be treated with two levels of stabilization^5,32^. Because of the rotation stress in patients with unilateral locked facet, the ligament and intervertebral disc are seriously damaged, as are accumulated adjacent segments. An injury to the adjacent segments may result in segmental instability during the long-term follow-up. Unfortunately, we could not identify a reliable factor for predicting adjacent joint instability in this study. However, the injuries of disc, ligamentous complex, and vertebra at adjacent segment can be easily observed on MRI examination. Overall, adjacent segment injury or instability in cases of UCLF should be considered during management.

## Conclusions

UCLF is a special form of unilateral lateral mass-facet injury that leads to severe injury of the intervertebral disc, ligament complex and vertebra of the cervical spine. Our results provide evidence that UCLF is a rotationally unstable cervical spine injury. The injury mechanism of type I seems to be attributed to flexion load, and it follows a rotation force with a smaller lateral tilt component. But a primary rotational and lateral tilt injury combined with a smaller flexion load may occur in type III. For the type II injury pattern, the mechanism of injury may be complex and diverse; distractive flexion injury is related to the type II A injury pattern, while hyperextension with a rotation injury may lead to the type II B or II C fracture pattern. The patients of type I without lateral mass-facet fracture had a worser and higher prevalence of spinal cord injury than that with fractures. Most of the patients with types II A and II C had nerve root compression in the neuroforamen resulting from fracture fragments of the superior articular process. The associated injury of disc, ligamentous complex, and vertebra had a significant difference among the types or subtypes, and the successful percentage of closed reduction in type I was significantly lower than that in type II and type III. The classification proposed in this study will contribute to understanding the injury mechanism, radiological characteristics, and neurological deficits in various types of UCLF, which will help the surgeons to evaluate the selection of surgical approach and fusion segment.
